# Germline genomic testing to assess the suitability of stem cell donors in the treatment of haematological malignancy: clinical ethics commentary

**DOI:** 10.1136/jme-2025-111111

**Published:** 2025-11-20

**Authors:** Helena Carley, Kate Sahan, Helen Hanson, Katie Snape, Sarah Westbury, Michael J Parker, Anneke M Lucassen

**Affiliations:** 1 South East Thames Regional Genetics Service, Guy’s Hospital, London, UK; 2 University of Oxford Nuffield Department of Medicine, Oxford, UK; 3 The Ethox Centre, Nuffield Department of Population Health, University of Oxford, Oxford, UK; 4 Peninsula Regional Genetics Service, Royal Devon University Healthcare NHS Foundation Trust, Exeter, UK; 5 Department of Clinical and Biomedical Sciences, University of Exeter Medical School, Exeter, UK; 6 South West Thames Centre for Genomics, St George’s University Hospitals NHS Foundation Trust, London, UK; 7 City St George’s, University of London, London, UK; 8 Translational Health Sciences, Bristol Medical School, Bristol, UK; 9 Department of Haematology, University Hospitals Bristol and Weston NHS Foundation Trust, Bristol, UK; 10 Oxford Clinical Genetics service, Oxford University Hospitals NHS Foundation Trust, Oxford, UK

**Keywords:** Ethics, Ethics- Medical

## Abstract

The increasing integration of genomic medicine into routine medical care brings to light issues of complexity and uncertainty in the interpretation of genetic variation.

We describe a fictional case study based on our experiences in the haematological malignancy setting to illustrate the complexities in using genomic testing to evaluate the suitability of relatives to act as stem cell donors. In particular, we demonstrate the ethical issues arising following the identification of a germline variant of uncertain significance (VUS) in a gene associated with inherited cancer susceptibility in a potential recipient. We discuss the factors that need to be considered, such as how this might influence donor selection, the potential for donor-derived leukaemia and the issues of offering testing to other family members.

Popular discourse suggests genomics can offer clarity and transformation in healthcare. This case highlights the ethically complex nature of decision-making in genetic medicine due to the familial aspects of genomic information, the uncertain nature of many genomic results and how changes in the use of genomic information in clinical practice can call for urgent decision-making. This highlights the need for increased interdisciplinary working which takes into account the factors driving decisions for each specialty and recommends access to independent ethics support for members of the multidisciplinary team.

## Introduction

 The last few years have seen genomic medicine integrated into the UK National Health Service (NHS), such that specialists can now request genetic tests directly relevant to their specialism. This move has highlighted two features which may not be reflected in the popular discourse about genomic medicine nor in some specialities’ postgraduate training: (a) the interpretation of genetic variants is complex and often uncertain and (b) that relatives are drawn into the management of patients and this complexity and uncertainty may also apply to them.^1^ Here, we use a fictionalised case study, based on real world experience in the context of stem cell donation for haematological malignancy to consider the ethical issues arising in clinical practice.

### Case

A young adult male patient, B, is referred urgently to his local clinical genetics service. B has no relevant medical history but his brother, A (also a young adult), has acute myeloid leukaemia^1^ and has been recommended to have an allogeneic haematopoietic stem cell transplantation (allo-HSCT) with curative intent. A’s medical history includes a rhabdomyosarcoma^2^ diagnosed at the age of 4 years which was successfully treated. In the family history, B and A’s mother, C, was diagnosed with breast cancer in her early 30s and died from her disease (figure 1).

**Figure 1:**
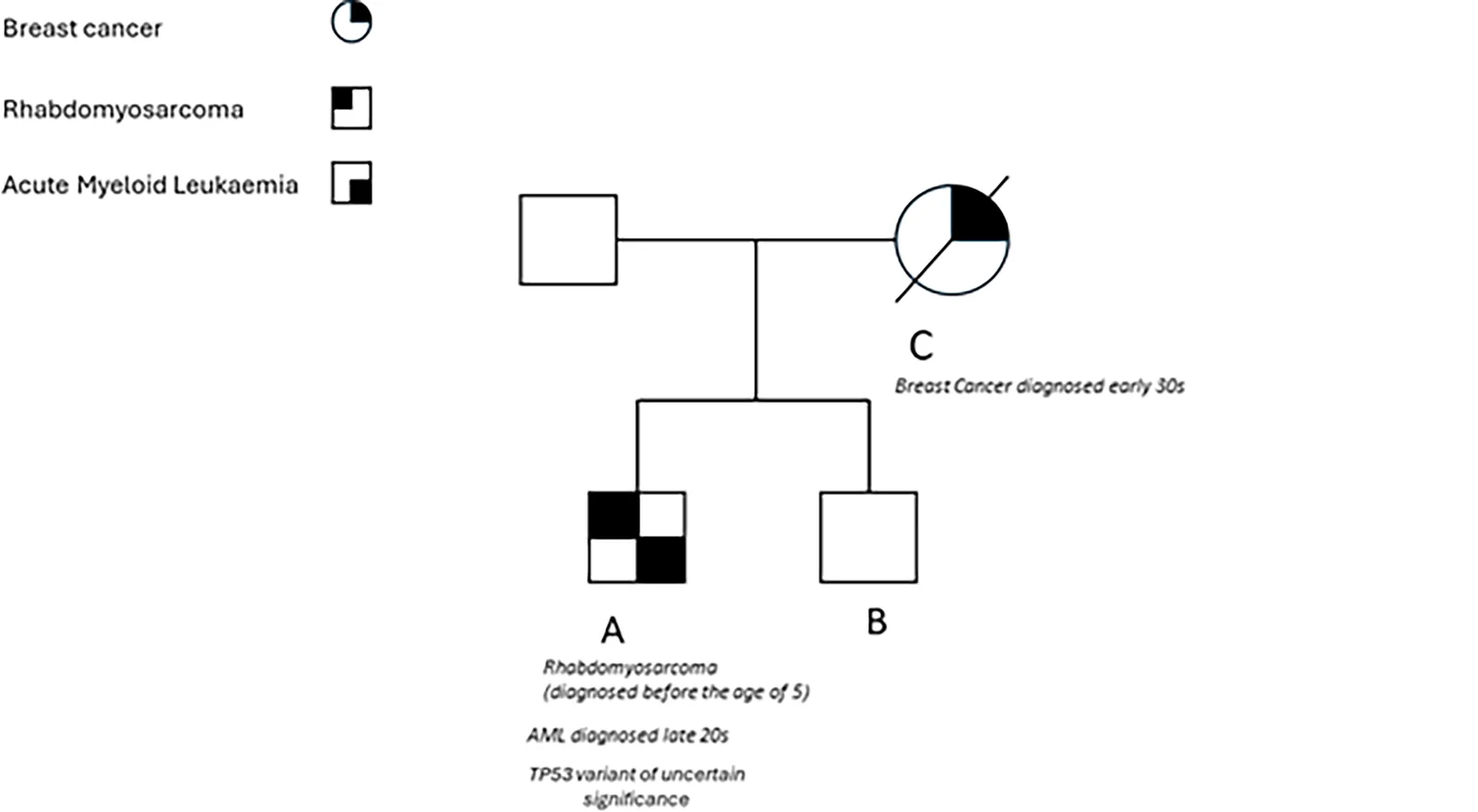
Pedigree. AML, acute myeloid leukaemia.

Paired tumour and germline whole genome sequencing (WGS) of A^3^ has detected a heterozygous germline variant of uncertain significance (VUS) in the *TP53* gene. Certain germline variants in *TP53* are associated with Li-Fraumeni Syndrome,^4^ a heritable condition associated with a very high lifetime chance of developing cancer including sarcomas, young onset breast cancer and haematological malignancies. There is a high clinical suspicion of Li-Fraumeni Syndrome in this family since A and his mother have had young-onset *TP53*-related cancers. However, the finding of a *TP53* VUS in A does not confirm this diagnosis. For instance, the predisposing variant may lie outside the areas of the *TP53* gene which have been analysed (eg, there may be a deep intronic variant affecting gene splicing, an alternative predisposing variant in a different gene or there may be no single predisposing variant in the family).

B is being considered as a stem cell donor for A. The haematology team wishes to pursue germline genetic testing for B to rule out the same *TP53* VUS. They argue that if detected, the VUS might preclude B from being a stem cell donor. If B also had a predisposition to cancer including leukaemia arising from Li-Fraumeni Syndrome, then donation from B could cause donor-derived leukaemia in A. The magnitude of this risk is not known but needs weighing against the likelihood of a successful transplant if using an unrelated donor and the possibility that the *TP53* VUS is not associated with Li-Fraumeni Syndrome, that is, that the variant is benign and unrelated to the cancers the family has experienced.^5^

## Use of genetic testing and allo-HSCT in the management of haematological malignancy

### The roles of somatic and germline genetic testing in diagnosing haematological malignancy

There is increasing recognition that haematological malignancy can arise on the background of a hereditary cancer predisposition syndrome.^2–4^ Testing of genes associated with a predisposition to haematological malignancy is increasingly available with more variants in relevant genes evaluated for their potential pathogenic role than previously.^5 6^ In the case above, paired testing of DNA from bone marrow containing leukaemic cells (somatic) and from skin cell tissue not involved in the malignancy (inferred germline) has been analysed and allows assessment of whether variants identified are present in the germline or only in the tumour.^6 6 7^ A variant identified in both is inherited; a variant identified only in the tumour is acquired and is likely not present in other cells in the body. The expanding use of paired WGS in cancer patients, alongside a growing recognition of how heritable components contribute to cancer development, means genetic testing can both inform prognosis and treatment options for patients and potentially alert biological relatives to their risks.

### Interpreting germline VUSs

It is often unclear whether a genetic variant predisposes to a disease (so-called ‘pathogenic’), even if found in a gene associated with the clinical condition in question. Each person harbours some 100 000 rare variants in their genome, most of which will play no role in such a condition but with little or no research findings to guide whether they might be pathogenic. Guidelines for gene variant classification have emerged to support the consistent interpretation of variants.^8^ The classification involves assigning ‘points’ to different pieces of evidence (eg, population, functional and phenotypic data) to provide a probability of variant pathogenicity. While the probability lies on a continuous scale, variants are assigned to one of five categories for the purpose of clinical decision-making (figure 2).

**Figure 2:**
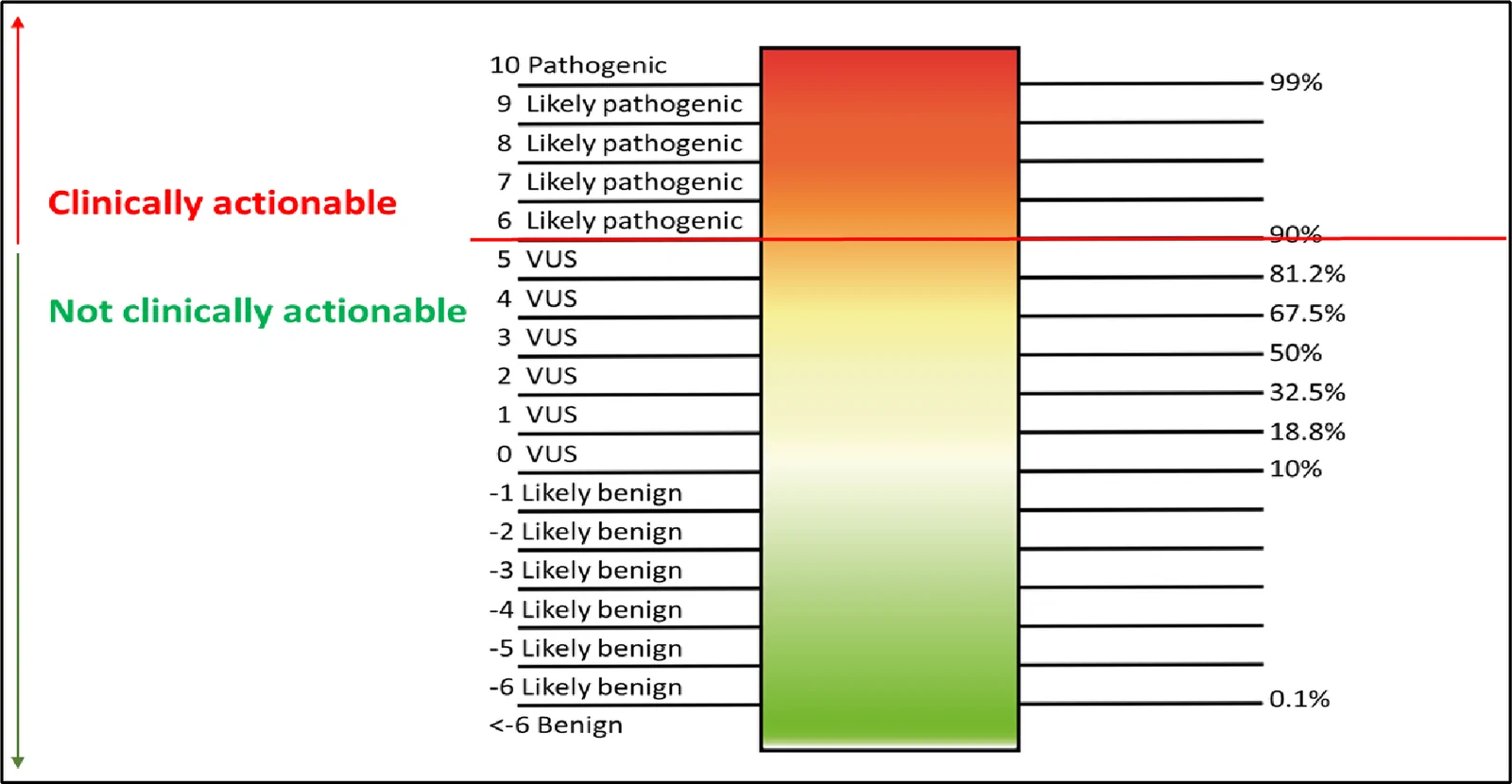
Variant classification heat map. This shows the points (derived from evidence in support of pathogenicity) required to classify a variant into one of five main categories. Different pieces of evidence for variant pathogenicity can be weighed together to give an evidence points score (−6 to 10) corresponding to a likelihood of pathogenicity. The breadth of the ‘uncertain significance’ category means that most variants will fall into this category. Clinical actionability occurs when a variant has a 90% chance of being pathogenic represented at six evidence points. Adapted from Figure 3.^9^ VUS, variant of uncertain significance.

For many variants, there is insufficient evidence to assign them to clearly benign or pathogenic categories. For those with a probability of pathogenicity between 10–90% a classification of uncertain significance (VUS) is made. With additional (often research) evidence, some VUS may be reclassified towards <10% (likely benign/benign) or >90% (likely pathogenic/pathogenic) categories. Because of this uncertainty, most VUS are no longer reported in patients’ records; however, those that score close to a 90% probability may be reported as these are most likely to be reclassified once more evidence is accumulated.^9^ The VUS classification is thus not normally used in clinical decision making: it does not trigger testing in other family members (unless segregation in a family could help variant classification) nor does it alter clinical management.

In the case above, the variant scores five evidence points, equating to ~81–90% likelihood of pathogenicity. Even with the clinical suspicion of Li-Fraumeni Syndrome due to the types of cancers within the family, this is insufficient^7^ to reclassify the *TP53* variant to one that could be used for clinical decision making or predictive testing in family members. Standard practice for genetic services might be to follow-up such families to see whether further evidence clarifies the pathogenicity—sometimes over several years. This is unhelpful here because a decision about stem cell donation must be made in weeks. Given that reclassification to improve predictive value in relatives is not possible in the short term, it is unclear whether to diverge from standard practice by testing B for the *TP53* VUS and what follows if he is found to be a carrier.

### Allo-HSCT in haematological malignancy

Allo-HSCT is a standard treatment for some haematological malignancy. Stem cells may be donated by biological relatives or unrelated donors or be obtained from cord blood banks. While donor selection can be complex, histocompatibility^8^ between donor and recipient is a key variable in transplant success. This is primarily assessed by donor-recipient matching of human leucocyte antigen (HLA) (also known as ‘tissue typing’), with HLA-identical siblings generally considered optimal donors (see Chapter 9 of The EBMT handbook^10^). The availability and accessibility of relatives may also make them preferable to apparently equivalently matched unrelated donors.

Potential donors may be excluded from donating if they have certain pre-existing health conditions to reduce the risk of stem cell donation harming the donor and/or the recipient. In the case above, the possibility of Li-Fraumeni Syndrome in this family suggests that B may also have inherited the condition and, if so, the risk of causing donor-derived leukaemia in A needs consideration. However, alongside current unknowns (B’s VUS status is currently unknown, Li-Fraumeni Syndrome in this family is suspected not diagnosed), there are also no data on the chance of donor-derived leukaemia in Li-Fraumeni Syndrome. That said, allo-HSCT is a high-risk procedure so other risks should be minimised. Yet, if this leaves A without a well-matched donor, such a cautious stance may be misguided.

Haematologists are highly experienced at interpreting complex and uncertain genetic data in the setting of somatic and germline testing. Yet, differences in perspective and clinical priorities may lead members of the multidisciplinary team (MDT) to hold different views on how to proceed. In this case, the haematology team have a primary responsibility to A and are driven by a need to make urgent treatment decisions for him, while the clinical genetics team have indirect involvement in A’s leukaemia care and are better placed to consider the longer-term implications of genetic testing for him and his family.

## Discussion

The case above raises complex ethical issues related to: (1) the familial nature of genetic predispositions to disease; (2) uncertainty about variant classification as genomic testing identifies more variants than targeted testing; (3) a need to make urgent treatment decisions with no time for gathering supporting evidence and (4) challenges for interdisciplinary and MDT working. While some of these issues are common to other applications of clinical genomics, their existence in the context of time-limited tissue donation decisions provokes particular challenges.

### Interdependency

Ethical challenges can arise in situations where one patient’s (urgent) care depends on information about the genetic status of others—often their relatives. This reflects the inherently familial nature of genomic medicine; we share much of our genetic makeup with our close relatives. While in other familial settings, A’s status would not trigger immediate testing in B, here the request is urgent and seen as necessary for A’s treatment. As a consequence, B has limited time to consider the pros and cons of testing. B’s offer of donation to his brother draws him in to an examination of his own future cancer risk, which he may not wish to know about (yet). This orientation in genomic medicine towards ‘unaffected’ relatives providing DNA samples to help an ‘affected’ patient means families are increasingly confronted with information relevant to their own health (see also the example of trio testing).^11^ If B is also at risk, then early detection and risk-reduction strategies are recommended because of the high lifetime risks of cancer.^12^ However, a VUS in the *TP53* gene would not be sufficient to receive such screening, so testing is not indicated on that basis. It seems inconsistent to advise B that his result will determine his suitability as a donor, but will not determine whether he is eligible for screening or reproductive risk counselling.

### Uncertainty

Genomics is associated with promises of clarity but the uncertain clinical relevance of genetic data often remains poorly understood.^13^ Uncertainty can be for reasons of scale or lack of context. For example, a whole genome sequence contains around 100 000 very rare variants, and each person has around 50 variants that have been described in the literature as pathogenic, often for severe childhood conditions that they clearly do not have as healthy adults.^14^ So too, knowing the context of a variant matters for its clinical interpretation. For example, if found in a person with a condition (or a family history of a condition) previously linked with a particular variant, the interpretation will be different from a finding within a healthy population screen. Additionally, some variants will only exert their effects given certain non-genetic factors (environment, lifestyle factors) or the presence of other genetic variants.

While variant classification guidelines exist, these cannot account for all contextual factors, so despite apparently precise probability criteria for variants, making judgements about their clinical relevance remains difficult. In the case above, the interpretation of the same variant might be different in another treatment context or with a family history less suggestive of an underlying haematological malignancy predisposition and might never be clinically reported.

A further point of uncertainty relates to how the clinical significance of variants can change as more evidence is accumulated.^15^ One study found that around 8% of VUS were reclassified over 1–2 years amended reporting time, with the majority reassigned to a category with a lower likelihood of being clinically relevant.^16 9^ While specialists requesting genetic tests should convey this possibility to patients, communication is complex.^15^ The very discussion of a finding might lead many to assume an actionable result of some sort—after all, why is it being reported if not?—leading to understandable caution from genetics professionals about turning data into information prematurely.^1^

To resolve uncertainty, we might do tissue typing in B, since if he is not a good match, there would be no indication for urgent germline testing. Yet even if B is an incompatible donor, the family history would still recommend B’s referral to clinical genetics to discuss his risks and explain why testing for the *TP53* VUS is not indicated. If B is compatible and does not have A’s *TP53* VUS, careful communication would need to ensure that this does not offer false reassurance of his own risk of developing Li-Fraumeni Syndrome. There may be other, as yet unidentified variants driving A’s disease which are shared with B. Therefore, a transplant from B could theoretically still result in an increased chance of A developing donor-derived leukaemia.

We have described this fictional case because of clinical situations where donors have been rejected on the basis of a VUS.^9^ Where a VUS has been reported, it lies close to the 90% probability of being pathogenic and thus the potential for reclassification needs careful consideration. Discussion within a specialist MDT setting which combines clinical and genomic variant expertise is important. Where testing for a VUS is made available for the purpose of informing donor selection, careful counselling is recommended to advise relatives of the associated uncertainties.^6^

### Urgency of requirement to make decisions in the absence of sufficient evidence

Genetic testing is increasingly requested to guide other urgent treatment decisions (eg, choice of breast surgery in *BRCA1/2* related breast cancer or deciding whether to terminate a pregnancy on the basis of prenatal genetic testing findings^17^). This can mean that when a variant is found, the supporting evidence required to adequately inform clinical decision-making will not have had sufficient time to accumulate.

In the case above, a key issue at stake is whether, due to lack of time to gather evidence to explore the significance of the identified *TP53* VUS, B (or any other relation to A) should be ‘worked up’ as a viable donor. In our case, taking the VUS as action-guiding forces practitioners to choose between either exercising over-caution (by excluding B as a donor) or accepting a possible risk by treating the variant as uncertain (proceeding with B as a viable donor). In both scenarios, variant classification fails to add much to the short-term decision about whether B is a suitable donor. Instead, it falls to individual or team responsibility to make a judgement call.

One way to resolve this might be to say that a VUS (unless particularly ‘hot’, see Section 7.1.1 of ^9^) should not be reported to clinicians in urgent settings as it might be misinterpreted. Another option would be to say that in haematological malignancy cases with a strong family history of cancer and no identified genetic variant cause, relatives might never be the safest donation option due to the possibility of an as yet unknown (shared) variant contributing to disease. Instead, unrelated donors would be prioritised. It is also important to recognise that donor registries may contain individuals with germline cancer predispositions which they are currently unaware of. Therefore, using an unrelated donor might still risk donor-derived disease.^6^

It could be that no other donors are available, and B represents the most likely chance of a cure for A. How would the family feel if B’s stem cell donation is denied? Limited options for treating A might mean that appreciations of risk start to differ among the various deciders or even that unknown risks are difficult factors to guide decision making. What might matter more, in the face of uncertain risks and limited options, is that B has tried to help his brother. This speaks to the importance of involving patients and families in decision-making, ensuring high quality communication of risk and uncertainty, but also helping relatives to identify the most relevant factors *to them* to make the decision.

Finally, there are also procedural and practical considerations relating to time and urgency, for example, reporting timeframes for clinical scientists and ensuring patients like B can be seen urgently in clinical genetics with corresponding implications for demand on services and workload. Additionally, healthcare professionals report urgency as adding to the challenges of ethical decision-making; they ‘do not have time to grapple with the ethics’.^18^ This highlights the importance of developing and evaluating effective models for situated ethical decision-making that empower health care professionals (HCPs) to arrive at justified decisions even under time pressure. This is particularly important given that features of the case above may limit the usefulness of guideline-driven approaches and wider MDT discussion may be required to reach a clinical decision. Together, this calls for approaches to decision-making which better reflect context, clinical need and clinical urgency.^1^ Any kind of contextual, situated approach depends on having ‘ethically prepared’ individuals and structures equipped to make this a regular feature of practice.^19^

### Challenges for interdisciplinary and MDT working

Underpinning many of the issues above are challenges relating to how interdisciplinary perspectives on cases can both enrich and complicate ethical decision-making. Perspectives in this case—from the fields of genetics, haematology, transplant and pathology—are formed by a range of factors including practitioners’ core specialism, their level of professional experience, knowledge of other specialisms relevant to the case, understanding and appetite for risk, comfort with uncertainty and degree of ethical decision-making support and experience. However much a practitioner is open to the norms of other specialisms, their practice will be rooted in their own training, clinical expertise and experience. This can make it professionally and psychologically challenging for two specialisms—one more used to certainty in the outcomes of treatment options and the other more comfortable with outcome uncertainty—to make ethical decisions together.

In light of these challenges, we suggest the following measures to support MDT decision-making in time-dependent haematology-oncology settings. First, it should be acknowledged that true MDT working—where clinical genetics, clinical scientists and haematologists are in the same meeting—is logistically hard to achieve yet essential to provide sufficient time, space and expertise to complex decision-making. Second, practitioners should be encouraged to understand the factors driving practice and differing priorities of other specialisms through collaborative working. This is especially important where opinions differ and a compromise must be reached to address conflicting approaches. While haematologists have long been familiar with the technical and familial aspects of genomic medicine (eg, in haemoglobinopathies and haemophilia), other specialisms may need training in the technical aspects of variant interpretation and accessing other relevant clinical data. Third, those involved in MDT discussions should have access to independent ethics support where more complex discussions around ethical decision-making are required beyond what can be offered by the clinical MDT. In the UK, NHS clinical ethics committees can act as rapid ethics case reviewers and can provide an independent perspective from individuals not directly involved in the patient’s care. This can be a valuable way to abstract from the complexity of context and professional responsibilities. However, the availability of ethics support is variable by trust and we suggest there should be greater planning and provision for ethics support for these types of urgent cases. An urgent ethics discussion is also possible independently of NHS ethics support services through groups such as the UK Genethics Forum (which can convene ad hoc meetings with key specialisms) in addition to their regular meetings three times a year.^20^ The particular value of this Forum is that it brings together genetics professionals from settings across the UK, and beyond, which enables both a diverse space for discussion and support and also the potential for learning across the system.

## Conclusion

We have described how germline genetic predisposition to haematological malignancy is identified more frequently through the routine genomic testing now offered to individuals affected with haematological malignancy and outcomes of this testing may help stratify potential stem cell donors. Often, the best and most readily available donors are found in families but they may share germline disease predispositions. How to treat the potential donor and the patient appropriately raises ethical issues relating to familial interdependency, uncertainty, and challenges of interdisciplinary working. These issues reflect challenges in classifying gene variants as clinically actionable when other contextual factors are only partially known. Such issues are more pressing and complex in haematological malignancy compared with other areas of medicine due to the urgent context of treatment using stem cell donation. We recommend close MDT working and rapid, flexible and situated ethics support for clinical geneticists and non-genetic specialists who need to consider germline findings in their practice. We also advise careful, contextually sensitive clinical genetics counselling for patients and families weighing up whether to use related donor stem cells for certain types of haematological malignancy.

## Data Availability

Data sharing not applicable as no datasets were generated and/or analysed for this study.

## Footnotes

A form of haematological malignancy or blood cancer.

Rhabdomyosarcoma is a type of soft tissue sarcoma, a rare cancer which is more common in children and adolescents.

Paired whole genome sequencing (WGS) in haematological malignancies compares gene variants present in tumour cells (typically bone marrow cells), with those present in a germline sample (non-tumour cells, typically a skin biopsy) to see which variants are somatic (occur only in the tumour) and which occur in the germline (and may be inherited). See Section 2.1.

Li-Fraumeni Syndrome is also known as heritable *TP53*-related cancer syndrome.

The risk of donor-derived leukaemia in A is considered the main reason to reject B were he to have Li-Fraumeni Syndrome, rather than risks to B himself. There is currently insufficient evidence that donor procedures involve increased risk for individuals with presymptomatic Li-Fraumeni Syndrome, so we have not added this as a reason to not accept B as a donor. This situation may change in the future.

i.e represent a somatic clonal event associated with the development of malignancy.

Except in limited situations where demonstration of segregation of a variant in other affected family members would aid in interpretation of a variant.

Compatibility between the tissues of different individuals.

And over a longer reporting time period, we might expect many more VUS to be re-classified.
